# The Fabrication of a Gellan Gum-Based Hydrogel Loaded With Magnesium Ions for the Synergistic Promotion of Skin Wound Healing

**DOI:** 10.3389/fbioe.2021.709679

**Published:** 2021-09-13

**Authors:** Wenqiang Li, Xingling Jian, Yanfen Zou, Lin Wu, Haiyan Huang, Hui Li, Dandan Hu, Bo Yu

**Affiliations:** ^1^ Department of Dermatology, Skin Research Institute of Peking University Shenzhen Hospital, Peking University Shenzhen Hospital, Shenzhen, China; ^2^ Guangdong Provincial Engineering Technology Research Center for Sports Assistive Devices, Guangzhou Sport University, Guangzhou, China; ^3^ Child Healthcare Department, Guangzhou Women’s and Children’s Medical Center, Guangzhou Medical University, Guangzhou, China

**Keywords:** gellan gum, magnesium ion, polyacrylamide, skin wounds, hydrogel

## Abstract

To accelerate serious skin burn wound healing in a convenient manner, an interpenetrating network of hydrogel consisting of gellan gum and polyacrylamide was synthesized by chemical crosslinking and Mg^2+^ ion immersion techniques. The prepared Mg^2+^@PAM/GG hydrogel was characterized by morphology, water vapor loss, swelling ratio, rheological properties, tensile mechanical, biocompatibility, and flow cytometry study. The results show that Mg^2+^@PAM/GG hydrogel’s mechanical strength could be enhanced by the dual network structure and physical crosslinking agent Mg^2+^ ions. In addition, the tension strength of Mg^2+^@PAM/GG hydrogel is obviously increased from 86 to 392 kPa, the elongation at break increased from 84 to 231%, and crosslinking density N increased from 4.3 to 7.2 mol/m^3^ compared with pure GG hydrogel. The cumulative release curve of Mg^2+^ ions shows that the multiple release mechanism of Mg^2+^ ions belong to non-Fick’s diffusion. Meanwhile, *in vitro* experiments show that Mg^2+^@PAM/GG double network hydrogel has favorable proliferation and an NF-κB pathway inhibition property for fibroblast cells. Finally, the healing effect of the Mg^2+^@PAM/GG was evaluated in a rat full-thickness burn model. The animal study demonstrates that Mg^2+^@PAM/GG could accelerate the healing efficiency in case of the sustained-released Mg^2+^ ions in wound beds. Considering this excellent performance, this convenient prepared hydrogel has great potential as a commercial application for skin full-thickness burn healing materials.

## Graphical Abstract

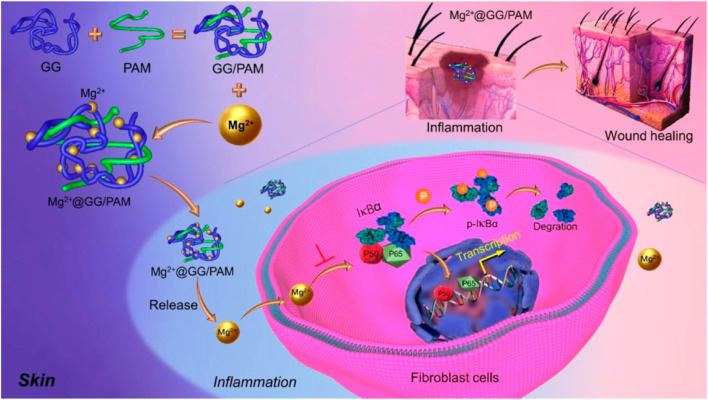

## Introduction

Burn wounds are serious devastating traumas with significant cost for both individuals and the health care system. Among all types of burns, third-degree burns, also known as full-thickness burns, destroy the full thickness of the skin, provoking immediate cell death and matrix destruction with the most devastating damage at the surface of the wound. Skin grafting and dressing is the traditional treatment for full-thickness burns but often with delayed wound healing, tissue necrosis, and scar formation. To solve the challenge associated with severe burns, various active hydrogel biomaterials have been developed to provide a beneficial environment for wound healing (Chouhan and Mandal, 2020; Wang et al., 2020).

An ideal hydrogel for severe burn repair should satisfy those requirements, include keeping the wound moist, absorbing excess exudate, covering the sensitive underlying tissue without adherence, inhibiting inflammation development, and actively accelerating the wound healing process at a low cost. Gellan gum (GG) is a kind of naturally anionic polysaccharide that is produced by the bacterium sphingomonas elodea (Zia et al., 2018). It is exploited in many commercial applications, including food additives, drug release systems (Palumbo et al., 2020), and emulsifying products (Ismail et al., 2019). Recently, Jian Yao Ng et al. developed an interpenetrating polymer network (IPN) hydrogel comprising GG, collagen, and adipose-derived stem cells (ADSCs) for the treatment of full-thickness burn wounds. The GG IPN hydrogels provide a cell-conducive environment for ADSCs, allowing the stem cells to be delivered to full-thickness wound beds (Ng et al., 2021). Huma et al. synthesized a GG-based hydrogel film with co-encapsulation of ofloxacin and lavender oil for the treatment of full-thickness wounds. The ofloxacin and oils were successfully incorporated into the hydrogel structure, moving in a controlled-release manner from the hydrogel to wound beds (Mahmood et al., 2021). Although those designed GG composite hydrogels possess favorable healing effects for full-thickness wounds, some disadvantages limit their commercial applications, such as the stem cell ethical challenge, chemical drug-doping approval problem, declined swelling behavior of the hydrogel, poor mechanical properties, or a relatively complex preparation process.

**SCHEME 1:**
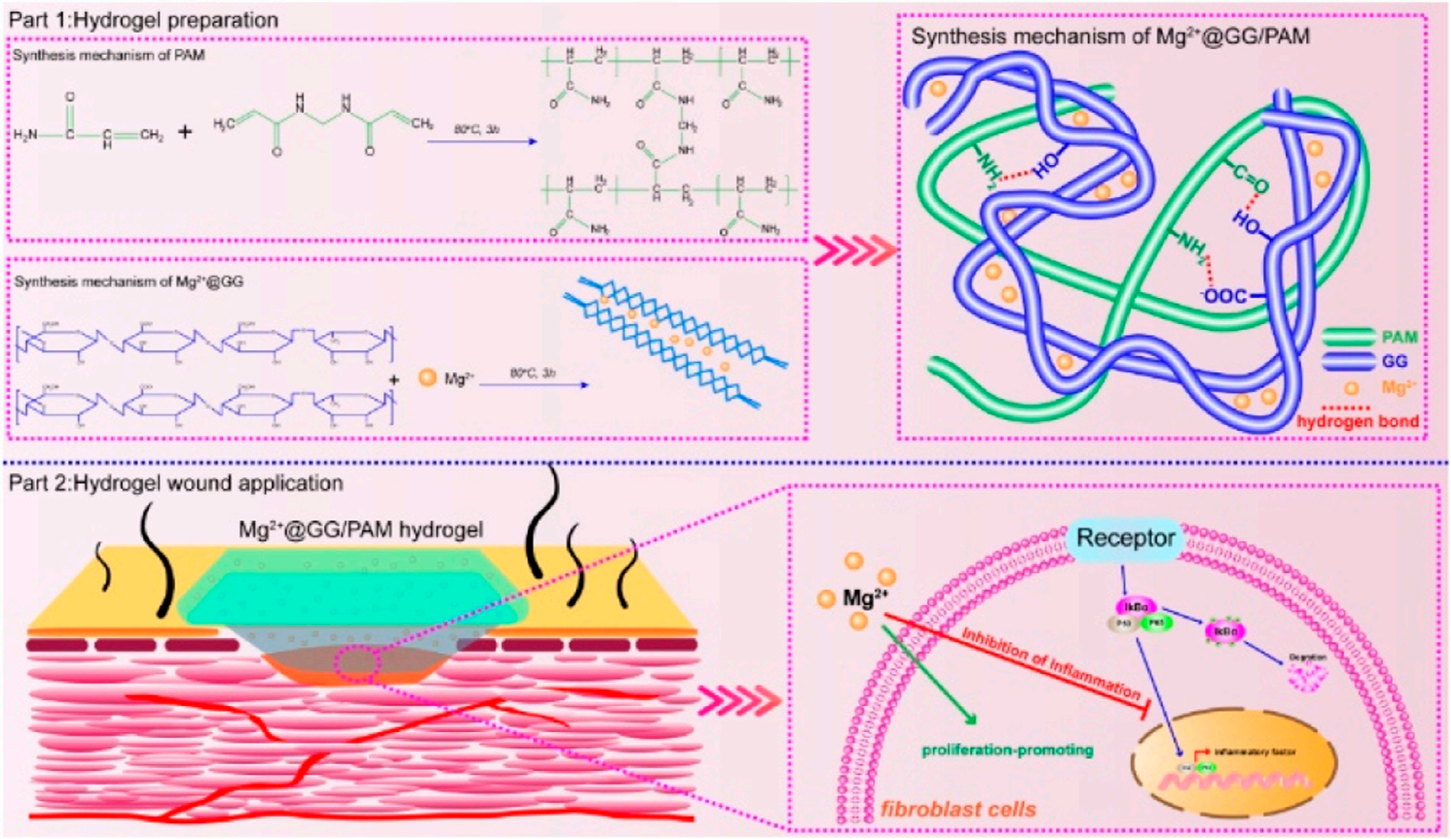
Schematic illustration of synthesis procedure for Mg^2+^@GG/PAM hydrogel and the repair mechanism of Mg^2+^ ions from Mg^2+^@GG/PAM hydrogel in the burn wound.

In the present work, we propose a convenient IPN hydrogel via chemical crosslinking and Mg^2+^ ion immersion techniques. This precursor hydrogel was prepared by heated acrylamide and GG monomer solutions, in which acrylamide was crosslinked by a chemical agent, and GG was crosslinked by metal Mg^2+^ ions, respectively. The poly-acrylamide (PAM) provides the high swelling ratio with good mechanical properties to make up the insufficiency of GG (Distler and Boccaccini, 2020; Randriantsilefisoa et al., 2020). More importantly, this dynamic dissociation and reassociation of the “GG-Mg^2+^” coordination bond enables the Mg^2+^ ions to be delivered into the burn wound in a stable and controlled manner. Magnesium ion acts in an enzymatic reaction for cell proliferation (Maier et al., 2004; Ma et al., 2016), cell differentiation (Wolf and Trapani, 2008), and collagen formation (Senni et al., 2003; Stechmiller, 2010; Coger et al., 2019), which effectively support the burn repair process. In this work, we focus on Mg^2+^ ion delivery behavior and evaluate the wound-healing effects of Mg^2+^ on a full-thickness wound model. As displayed in Scheme 1, Mg^2+^@GG/PAM hydrogel can effectively accelerate burn wound repair by promotion of fibroblast cell proliferation and anti-inflammation by releasing Mg^2+^ ions.

## Experiment

### Materials

GG (Gelzan^TM^ CM, G1910), MgCl_2_ (M8266), acrylamide (A8887), N,N′-methylenediacrylamide (MBAm, 1,108,970,050), and ammonium persulfate (APS, A3678) were purchased from Sigma-Aldrich. All reagents were purchased from Sigma-Aldrich without further purification.

### Synthesis of Mg^2+^@Gellan Gum/Poly-Acrylamide Hydrogel

The interpenetrating network hydrogel of GG and PAM was synthesized by mixing the following homogeneous ingredients: The synthesis of Mg^2+^@GG/PAM hydrogel was prepared by mixing the following ingredients in deionized water: 1.5 wt% GG, 1.5 mol/L acrylamide monomer, 0.015 mol/L MBAm, 5 mmol/L APS. In this solution system, MBAm served as a crosslinker, and APS served as an initiator. The GG/PAM hydrogel was synthesized at 80°C for 3 h by free radical polymerization. After polymerization, the hydrogel was washed in deionized water for 3 days at 10 °C to remove the residual monomer and micromolecules. The washed hydrogel was freeze-dried and immersed into Mg^2+^ ion solution (the concentration is 0.1 mol/L) for 12 h to obtain the Mg^2+^@GG/PAM IPN hydrogel. Finally, the Mg^2+^@GG/PAM hydrogel was freeze-dried for future use.

To validate the subsequent performance of Mg^2+^@GG/PAM hydrogel, pure GG hydrogels were also prepared by adding equal weight GG (equal to the quality sum of 1.5 wt% GG, 1.5 mol/L acrylamide, 0.015 mol/L MBAm, 5 mmol/L APS) in deionized water.

### Character of Mg^2+^@Gellan Gum/Poly-Acrylamide Hydrogel

FT-IR: The FT-IR spectra of hydrogels (lyophilized status) were measured using a Bruker instrument. Spectra ranged from 4,000 to 500 cm^−1^. The wave number resolution was selected as 2 cm^−1^.

Scanning electron microscopy (SEM) and energy dispersive spectrometer (EDS) analysis: The cross-section morphology of Mg^2+^@GG/PAM hydrogel (lyophilized status) was observed by SEM (Philips LEO1530 VPSEM). The chemical element distribution of Mg^2+^@GG/PAM hydrogel was analyzed by EDS.

Thermo gravimetric analysis (TGA): TGA (Mettler-Toledo) of hydrogels (lyophilized status) was executed with the temperature ranging from room temperature to 700°C with a heating rate of 10°C/min under N^2^ atm.

Tension tests: The hydrogels (hydrogel status) tensile property was executed by a universal testing instrument (UTM-Q422, Chende Jinjian Testing Instrument Co., Ltd., China) with a strain rate of 50 mm/min.

Rheological test: The rheological tests of hydrogels (hydrogel status) were executed through a rotational rheomete (DHR, TA Instruments, United States) operated in 20 mm parallel plate geometry and 1 mm gap distance. First, the gelation behavior of the hydrogel was tested via frequency sweep with a constant strain of 1% by varying amplitude frequency 0.1–100 rad/s. Finally, the strain sweep was tested with the constant frequency of 6.3 rad/s and oscillatory strain from 0.1 to 100%.

### Water Vapor Transmission Rate

The moisture permeability of Mg^2+^@GG/PAM hydrogel was conducted by the water vapor transmission rate (WVTR), and the detailed operation referred to ASTM standard E96–00 (ASTM E96-00, 2000). Briefly, the hydrogel was placed on the mouth of a cylindrical glass bottle (diameter 40 mm) containing a certain volume of deionized water and fastened to the bottle mouth edge to prevent water vapor fleeing away from the edge. The bottle was placed in a 37°C and 35% humidity environment for 24 h. The curve of weight loss versus time was recorded and plotted. From the slope of the curve, WVTR was calculated by the following equation:

$$WVTR=\frac{slope\times 24}{A}g/m^{2}/day$$

in which, A is the hydrogel test area with units m^2^.

### Swelling Measurement

Mg^2+^@GG/PAM hydrogel was freeze-dried and immersed in PBS solution at 37°C for 24 h. At a predetermined time, hydrogels were removed from excess PBS solution and weighed. Swelling ratio is calculated with the following formula:

$$Swelling\;\left(\%\right)=\frac{W_{w}\pm W_{d}}{W_{d}}$$

W_w_ is the swelled hydrogel weight in PBS, and W_d_ is the freeze-dried hydrogel weight.

### Study of Mg^2+^ Release Properties

Mg^2+^@GG/PAM hydrogel was immersed in 5 ml of PBS solution (pH = 7.4), and the solution was placed under 120 rpm shaking at 37°C for 64 days. At predetermined time points, 4 ml of solution was taken out to measure the Mg^2+^ release amount, and 4 ml volume of fresh PBS was added. The amount of released Mg^2+^ was determined using inductively coupled plasma-mass spectrometry (ICP-MS). The cumulative release of Mg^2+^ was calculated with the following equation: Mg^2+^ (%) = (total release of Mg^2+^/total load of Mg^2+^ in the sample) × 100%.

### Cell Culture

The viability and proliferation performance of Mg^2+^@GG/PAM hydrogel was tested using 3T3 mouse fibroblast cells and RAW264.7 cells. For 3T3 mouse fibroblast cells, the DMEM medium supplemented with 10% calf bovine serum was used as the culture medium. The fibroblast cells (1×10^5^ cells per well) were cultured onto the Mg^2+^@GG/PAM hydrogel (lyophilized status) surface for 1, 2, and 3 days. At day 3, acridine orange/ethidium bromide (AO/EB, Sigma Aldrich) was stained and observed by fluorescence microscope (Olympus TH4-200). Meanwhile, the proliferation ability of 3T3 fibroblast cells was quantified by the MTT (3-(4,5-dimethylthiazol-2-yl)-2,5-diphenyltetrazolium bromide) method. The control, GG, GG/PAM, and Mg^2+^@GG/PAM groups were added with 50 μL MTT solution and incubated 4 h. Then, the absorbance was detected using a microplate reader. The apoptosis of fibroblast cells was detected with flow cytometry by Annexin V-FITC/PI apoptosis kit (eBioscience, Thermo Fisher). For RAW264.7 cells (1 × 10^5^ cells per well), they were cultured in DMEM high-sugar medium onto a plastic plate or Mg^2+^@GG/PAM hydrogel surface for 72 h to study their anti-inflammatory ability. In addition, lipopolysaccharide (LPS) and PBS were added into the medium to make the concentration 10 mg/ml, respectively. In total, the cells were divided into four groups: control (PBS), LPS, Mg^2+^@GG/PAM (cultured onto Mg^2+^@GG/PAM hydrogel), and LPS + Mg^2+^@GG/PAM (cultured onto Mg^2+^@GG/PAM hydrogel with LPS added) groups. Then, they were collected, and we conducted the Western blot experiment.

### Western Blot Assays

For protein analysis, fibroblast cells were rinsed in PBS and lysed using radioimmunoprecipitation assay (RIPA) buffer containing 1% (v/v) phenylmethylsulfonyl fluoride (PMSF, P7626; Sigma-Aldrich). Afterward, the protein supernatant was harvested and detected by the bicinchoninic acid (BCA) protein assay kit (ab102536; Abcam, Cambridge, United Kingdom). The protein was denatured in a water bath (at 95°C, for 5 min) after adding a protein-loading buffer. The cells lysates were then processed with 12% SDS-PAGE gel electrophoresis at 120 V for 1 h. The polyvinylidene fluoride (PVDF) membrane (Millipore, Billerica, MA) was used to load proteins. Then Western blocking buffer was used to treat the PVDF membranes, which were incubated by anti-NF-κB p65 (ab16502) and β-actin (ab115777) primary antibodies overnight at 4°C. The next day, the tris-buffered saline (TBS) containing 0.1% Tween 20 (TBST) buffer was used to rinse the PVDF membranes twice, and then the PVDF membranes were incubated for 2 h by the secondary antibodies (1:2000; Proteintech, Rosemont, IL) in a Western secondary antibody dilution buffer. The Tannon 5,200 Multi image analysis system (Tanon Technology Co., Shanghai, China) was used to test all blot intensities. The Quantity One software (Bio-Rad, Hercules, CA) was used to test band density, and blots were carried out in triplicate.

### *In Vivo* Animal Test and Surgical Procedures

All animal experimental precepts conformed to the oversight of Guangzhou sport university laboratory animal ethics, Guangzhou, China (20,190,912,814,223). The 32 Sprague–Dawley rats (male, 200–300 g) were equally divided into four groups and given pentobarbital sodium through intraperitoneal injection, and the dorsal hair was removed. Deep burn wounds were generated on the dorsal skin using a hot nummular copper block with diameter of 18 mm, which was heated in a 100°C water bath. The copper block was placed on the shaven posterior-dorsum of each rat for 50 s to create a full-thickness burn model. Then, the polypropylene isolation chamber was implanted onto the wounds to inhibit skin shrinkage. The carnosus membrane of wound tissue was removed 24 h later. Then, the deep-thickness wounds were covered with GG, GG/PAM, Mg^2+^@GG/PAM hydrogel. The hydrogels were sterilized by Co-60 irradiation at 10 kGy (Guangdong Leida Technology Co., Ltd.) before treatment, and antiseptic gauze was placed as a cover over the hydrogels and sewn to the wound to prevent infection. Then, the wound was fastened with an elastic bandage to prevent wound contraction. The animal tissue specimens were obtained after treatment for 7, 14, 21, and 28 days.

### Histology Evaluation

The tissue surrounding the wounds was obtained 7, 14, 21, and 28 days after implantation of hydrogels. The tissues were fixed with paraformaldehyde for 24 h at 4°C, and hematoxylin-eosin (H&E) staining, Masson’s trichrome staining, and TNF-α immunohistochemical staining were carried out to evaluate wound healing and inflammatory reactions. The histological sections were examined using a stereomicroscope (Stereo Discovery, Zeiss). All samples were performed with at least three wounds per group. For immunohistochemical staining, the tissue paraffin sections were soaked in xylene solution for 15 min; soaked in 70, 80, 90, 95, and 100% alcohol solution for dehydration for 10 min each; and then PBS was used to wash off the extra alcohol. Then, 3% bovine serum albumin was used for nonspecific blocking for 40 min. Anti-TNF-ɑ primary antibody (TNF-ɑ, Abcam) was incubated overnight and rinsed with PBS buffer three times and then stained with secondary antibody at 37°C for 30 min, then rinsed with 0.01 M PBS buffer three times, about 5 min each time. Finally, a 3,3′-diaminobenzidine hydrochloride (DAB) color reagent kit was used to visualize color.

### Statistical Analysis

All tests were repeated in triplicate unless otherwise stated. Data are represented as average ±standard deviation (SD). Statistical analysis was conducted using analysis of variance (ANOVA) test with GraphPad Prism v8 software. *p* value was regarded as significant with *p < 0.05, **p < 0.01.

## Results and Discussion

### Morphologies and Properties of Mg^2+^@Gellan Gum/Poly-Acrylamide Hydrogel

**FIGURE 1:**
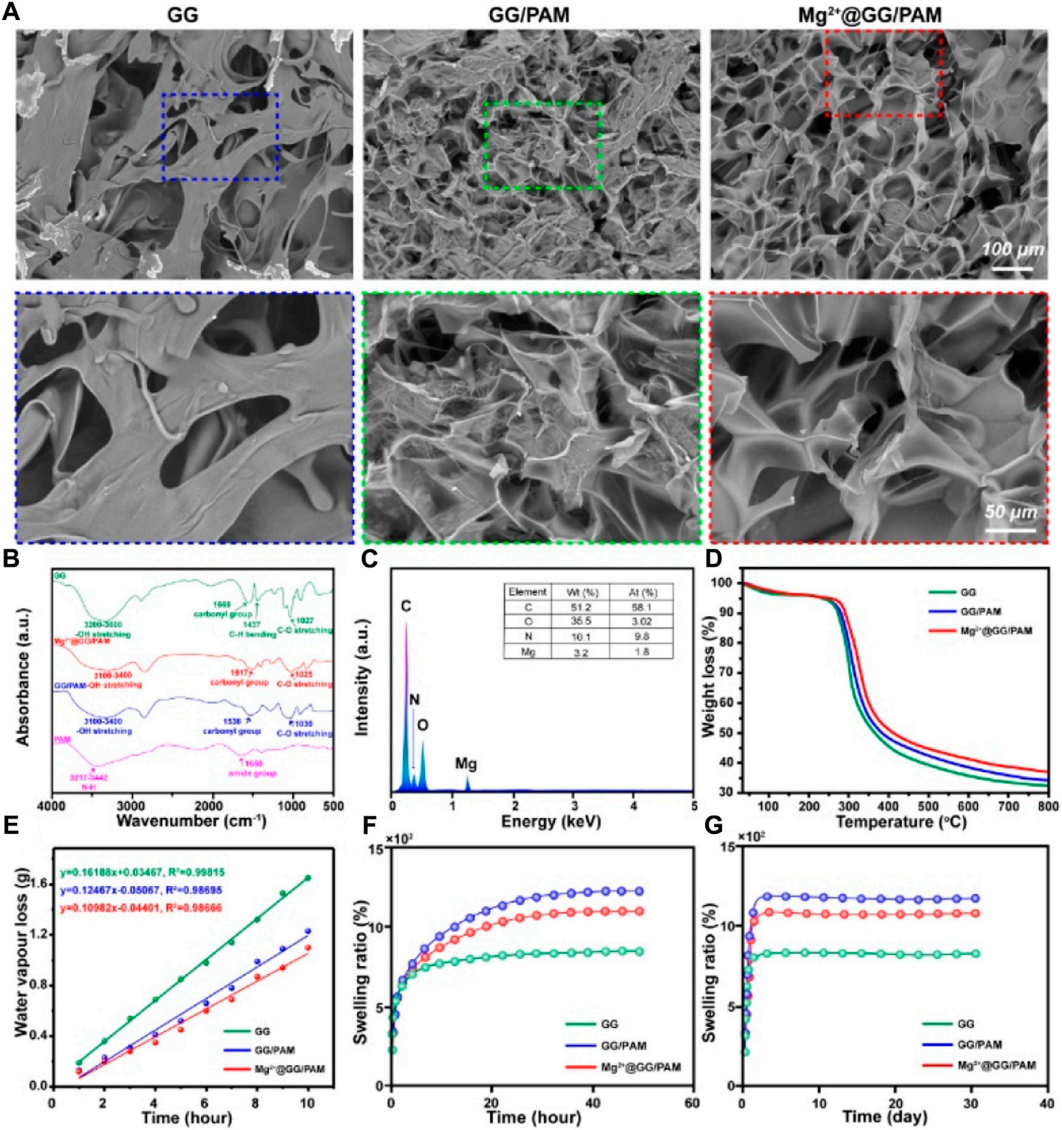
Characterization of Mg^2+^@GG/PAM hydrogel. **(A)** SEM **(B)** FT-IR **(C)** EDS analysis **(D)** TGA **(E)** Water vapor transmission rate **(F,G)** Swelling ratio.

GG and PAM hydrogel both served as a promising dressing biomaterial that have been widely considered in wound repair due to their biodegradability, chemical modification, nontoxicity, high water absorption, and good mechanical property. Here, a GG molecular chain was physically crosslinked by Mg^2+^ ions, and the acrylamide molecular chain was chemically crosslinked by N,N′-methylenediacrylamide to form a Mg^2+^@GG/PAM INP hydrogel. The cross-section morphologies of Mg^2+^@GG/PAM hydrogel were observed by SEM (Figure 1A). Mg^2+^@GG/PAM hydrogel showed an interconnected porous structure without obvious Mg nanoparticle residues that favored gas permeation and Mg^2+^ delivery. The synthesis of Mg^2+^@GG/PAM hydrogel was confirmed by FT-IR spectroscopy. The pure GG showed broad–OH stretching peaks ranging from 3,200 to 3,600 cm^−1^, C–O stretching at 1,027 cm^−1^, carbonyl group at 1,669 cm^−1^, and CH bending at 1,437 cm^−1^. The PAM hydrogel displayed an N-H stretching peak at 3,217–3,442 cm^−1^ and amide group at 1,650 cm^−1^. The GG/PAM composite hydrogel showed less sharp peaks for pure GG hydrogel indicating the hydrogen bond formed between GG and PAM. In addition, the carbonyl group and C-O stretching peak appeared to have bathochromic shift for the formation of hydrogen bond that equalizes the electron cloud density and, thus, reduced the stretching vibration frequency. In the spectrum of the Mg^2+^@GG/PAM group, the same peak shifted to a higher absorption frequency at 1,517 cm^−1^, and this indicates ionic interaction between the carbonyl group and Mg^2+^ ions. The EDS analysis is shown in Figure 1B; it can be seen that the Mg^2+^ element was detected at the concentration of 3.2 wt%, indicating that the Mg^2+^ ion was dispersed into the Mg^2+^@GG/PAM hydrogel. The TGA data is shown in Figure 1C; the crosslinking agent of the Mg^2+^ ions could reduce the thermal decomposition rate of the composite hydrogel. Additionally, an ideal wound healing hydrogel must keep the water loss speed at an optimal rate to make the wound moist and prevent the hydrogel from adhering onto the burn wound. The ASTM standard E96–00 was used to evaluate the hydrogels’ moisture permeability. Vapor loss of wound dressing ranging from 2000 to 2,500 g/m^2^/day is recommended for adequate moisture without wound dehydration (Queen et al., 1987). Based on the plot slope (Figure 1D), the WVTR of GG/PAM and Mg^2+^@GG/PAM hydrogel were calculated to be ∼2,382 and ∼2098 g/m^2^/day, respectively. Evidently, the WVTR of pure GG hydrogel exceed the standard and risked fast water loss in wound, and the Mg^2+^@GG/PAM hydrogel possessed a suitable WVTR to maintain a proper fluid balance in the wound area. Besides appropriate physical structure and vapor loss rate, the equilibrium swelling ratio (ESR) is also crucial for wound dressing. As the wound exudate is often generated from the wound surface, timely absorption of the exudate is important to maintain a moist environment and accelerate the wound healing rate (Yu et al., 2006). The ESR test **(** Figures 1E,F) showed that all the GG/PAM and Mg^2+^@GG/PAM hydrogels had a high water equilibrium swelling ratio more than 1,000% compared with the pure GG hydrogel, indicating that the hydrogels possess a good fluid uptake capacity, which is important for absorbing the excess edema fluid. The higher ESR in GG/PAM group is thanks to the high hydrophilic acrylamide. It is worth mentioning that the ESR slightly decreased in the Mg^2+^@GG/PAM hydrogel because of the increased crosslinking density by the Mg^2+^ ions.

### Mechanical Property of Mg^2+^@Gellan Gum/Poly-Acrylamide Hydrogel

**FIGURE 2:**
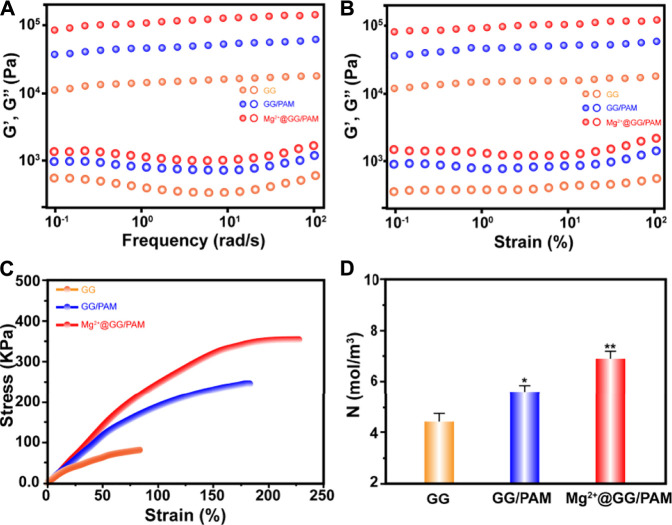
Frequency dependence **(A)** and strain dependence **(B)** of storage modulus (G′) and loss modulus (G″), tensile stress–strain curves **(C)**, corresponding crosslinking density N as a function of Mg^2+^@GG/PAM hydrogels **(D)**. The values are represented as mean ± SD (*n* = 3). *p < 0.05, **p < 0.01 vs control.

Good mechanical property is the guarantee for the dressing’s commercial application. The mechanical properties, including rheological property and stress–strain behavior of pure GG and Mg^2+^@GG/PAM IPN hydrogel, were tested. The angular frequency ω (Figure 2A) and strain (Figure 2B) depended on the storage modulus G′ and loss modulus G″ of the GG, GG/PAM, and Mg^2+^@GG/PAM hydrogels that were tested. G′ is always higher than G″ among the frequency range, and the G′ appeared to plateau in a low frequency range for all four hydrogels. This indicates that crosslinked networks have already formed in these hydrogels. In addition, both the G′ and G″ levels are highest in the Mg^2+^@GG/PAM hydrogel group, indicating that the Mg^2+^ ions could enhance the rheology mechanical property. The stress–strain curve is shown in Figure 2C; compared with pure GG hydrogel, the tension strength of Mg^2+^@PAM/GG hydrogel is obviously increased from 86 to 392 kPa, and the elongation at break increased from 84 to 231%. The enhanced Young’s modulus, breaking stress and strain of Mg^2+^@GG/PAM hydrogel, is because of the existence of the Mg^2+^ physical crosslinked GG and the MBAm chemical crosslinked PAM. What is more, PAM contains a polar amide group and hydroxyl group, and the GG chain contains hydroxyl groups, which can interact with each other to form hydrogen bonds to future enhance the mechanical property. To validate the crosslinking effect of this composite INP hydrogel, the crosslinking density N (Xiong et al., 2008) is calculated in Figure 2D. The crosslinking density of Mg^2+^@GG/PAM hydrogel is 7.2 mol/m^3^ compared with pure GG hydrogel (4.4 mol/m^3^). The good mechanical property of the Mg^2+^@GG/PAM IPN hydrogel is because of the dual network architecture creating the nonlinear tension against deformation, and the structure is similar to biological soft tissues.

### Mg^2+^ Ion Release Behavior and Hydrogel Degradation

**FIGURE 3:**
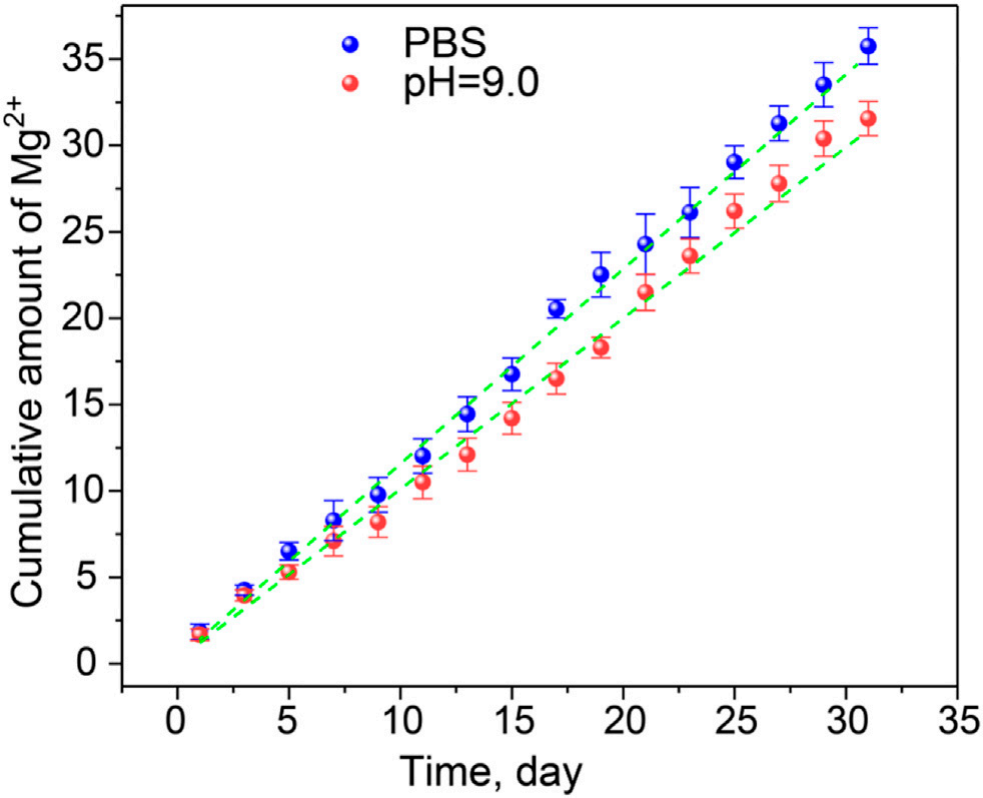
The release curve of Mg^2+^ from Mg^2+^@GG/PAM hydrogel in PBS (blue dotted), pH = 9.0 solution (red dotted), and Korsmeyer–Peppas fit curve (green dotted line). The values are represented as mean ± SD (*n* = 4).

The Mg^2+^ ion cumulative release curve at the predetermined time under PBS and pH = 9.0 conditions simulates the slightly alkaline environment in the burn wound. It can be seen from Figure 3 that the Mg^2+^@GG/PAM hydrogel group showed a controlled cumulative release rate of Mg^2+^ ions in the PBS and alkaline simulative condition. The release mechanism was brought by the ion exchange and the swelling property of the hydrogel. The release experiments show that Mg^2+^ shows better release performance without initial burst release. This is because Mg^2+^ ions act as the crosslinking agent of the GG molecular chain versus burst release and swelling. The Mg^2+^@GG/PAM hydrogel showed the best release performance with a cumulative release rate of 36.8 ± 1.95% in PBS and 32.1 ± 1.86% in pH = 9.0 at 31 days. The slightly declined cumulative release rate in pH = 9.0 might be the cause of the enhanced negative-charged carboxyl group, which future strengthened the interaction between the carboxyl group and Mg^2+^ ions. To better analyze the release behavior of Mg^2+^, the zero order release equation, first order equation, Higuchi equation, and Korsmeyer–Peppas model were fitted. The curve-fitting degree was calculated by the regression coefficient (*R* ^2^), which is listed in Table 1. All the *R* ^2^ value of the four models are higher than 0.9, and *n* was more than 0.45 in both PBS and alkaline solutions. The results suggest the multiple release mechanism of Mg^2+^ ions in the simulated environment. In addition, the value of *R* ^2^ for Korsmeyer–Peppas model is 0.99684, 0.99327, and *n* is 0.940, 0.979 in PBS and alkaline solutions, respectively. This result indicates that Mg^2+^ release performance features the drug diffusion and frame erosion release mechanisms and belongs to non-Fick’s diffusion *in vitro*. This release mechanism might be because Mg^2+^ ions participate in the skeleton of Mg^2+^@GG/PAM hydrogel. The good release behavior provides a guarantee for Mg^2+^@GG/PAM hydrogel to maintain biological activity ions for curing skin burn wounds.

**TABLE 1:** The release kinetic models of Mg^2+^@GG/PAM hydrogel.

Drug	Medium	*R* ^2^				*n*
Mg^2+^	PBS	Zero-order	First-order	Higuchi	Korsmeyer-peppas	
		0.99795	0.99489	0.95973	0.99684	0.940
	pH = 9.0	0.99511	0.99321	0.94412	0.99327	0.979

### Fibroblast Cells Cultured on the Mg^2+^@Gellan Gum/Poly-Acrylamide Hydrogel

**FIGURE 4:**
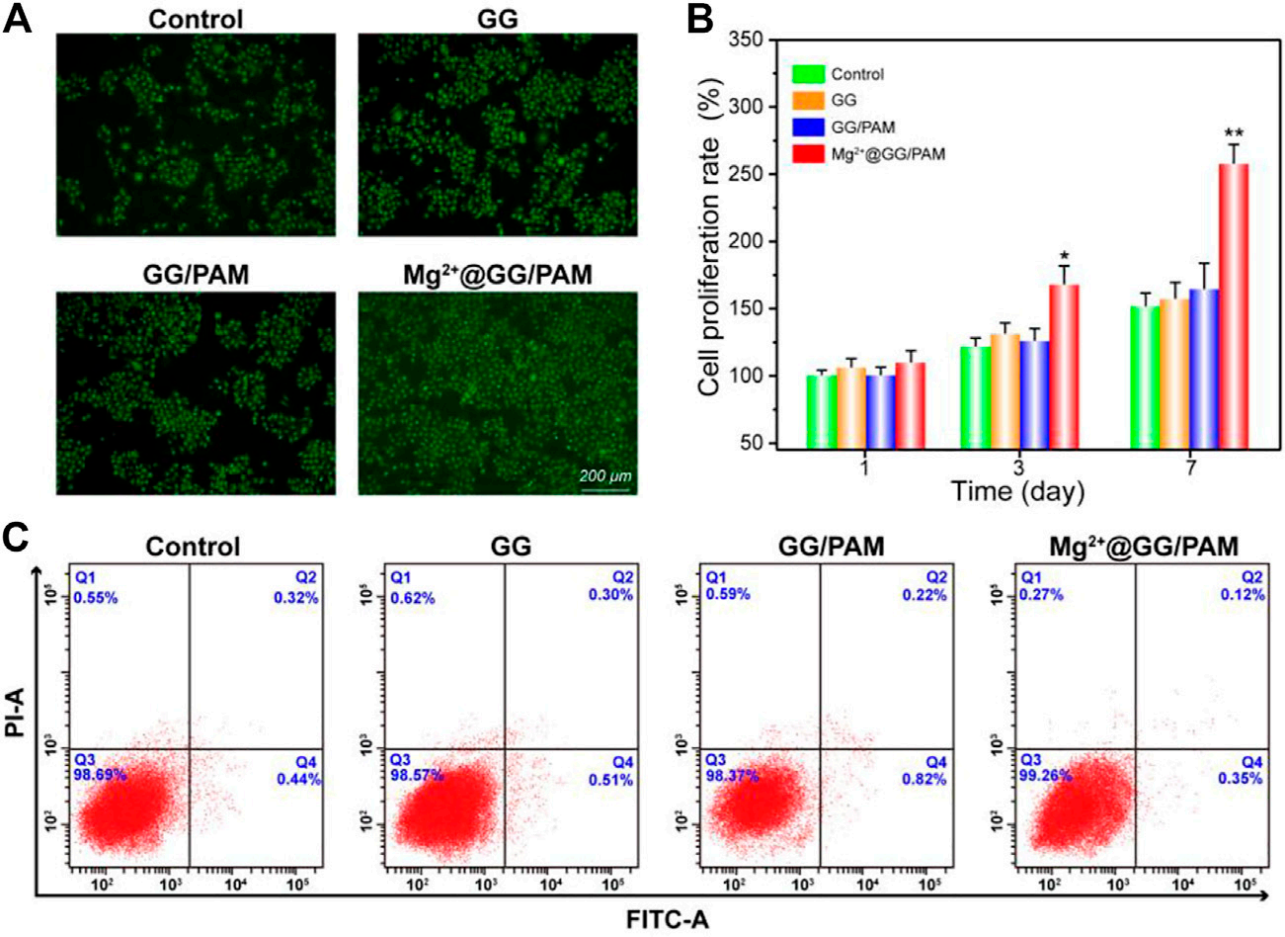
Cytotoxicity of Mg^2+^@GG/PAM hydrogel *in vitro*. **(A)** The AO-EB staining of fibroblast cells cultured with control, GG, GG/PAM, and Mg^2+^@GG/PAM hydrogel surface for 72 h. **(B)** The cell viability of fibroblast cells using ImageJ software for 1, 3, and 7 days (means ± SD, *n* = 4). **(C)** Apoptosis analysis of fibroblast cells by flow cytometer. *p < 0.05, **p < 0.01 vs control.

To validate the function of Mg^2+^@GG/PAM hydrogel in skin cell growth and proliferation, fibroblast cells were incubated on the Mg^2+^@GG/PAM hydrogel surface for 72 h and then observed by fluorescence microscope. Figure 4A shows the AO-EB staining of the fibroblast cells on the composite hydrogels. AO dye can penetrate the living cells and traverse an intact cytomembrane and embed into DNA, making the nucleus present with a green color. EB cannot penetrate the living cells, but it can access the dead cell nuclei and membrane. Therefore, the late apoptosis and dead cells can be stained with a bright red color (Yang et al., 2016). After culture for 72 h, the fibroblast cells cultured on the GG, GG/PAM, and Mg^2+^@GG/PAM hydrogels were homo-dispersed and visualized a green fluorescence. This shows that the Mg^2+^@GG/PAM hydrogels possessed favorable biocompatibility for fibroblast cells, which is essential for cell growth and proliferation (Li et al., 2020). Moreover, the cells cultured on the Mg^2+^@GG/PAM hydrogel had better proliferation ability compared with the other groups. The enhancement mechanism of cell proliferation may result from the presence of Mg^2+^ active ions. According to previous literature, the magnesium content is directly correlated to proliferation in normal cells as Mg ions stimulate DNA and protein synthesis. Mg deprivation, in turn, induces inhibition of DNA and protein synthesis, thus promoting growth arrest (Magnesium in cell proliferation and differentiation). Based on AO-EB fluorescence microscope images, we also calculated the cell proliferation amounts using ImageJ software (shown in Figure 4B). Clearly, the amount of fibroblast cells on the surface of the Mg^2+^@GG/PAM hydrogel increased significantly after culture at 3 and 7 days. To investigate the good biocompatibility effect of Mg^2+^@GG/PAM hydrogel for fibroblast cells, an apoptosis assay was analyzed. Figure 4C shows the apoptosis effect of fibroblast cells cultured onto control, GG, GG/PAM, and Mg^2+^@GG/PAM for 72 h detected by flow cytometer. Q2 and Q4 quadrants represent late and early apoptosis, respectively. The cells apoptosis ratio in the control, GG, GG/PAM, and Mg^2+^@GG/PAM groups is very little. This result further confirms that Mg^2+^@GG/PAM hydrogel possesses good biocompatibility for fibroblast cells.

### Mg^2+^@Gellan Gum/Poly-Acrylamide Hydrogel Inhibits NF-κB Pathway in RAW264.7 Cells

**FIGURE 5:**
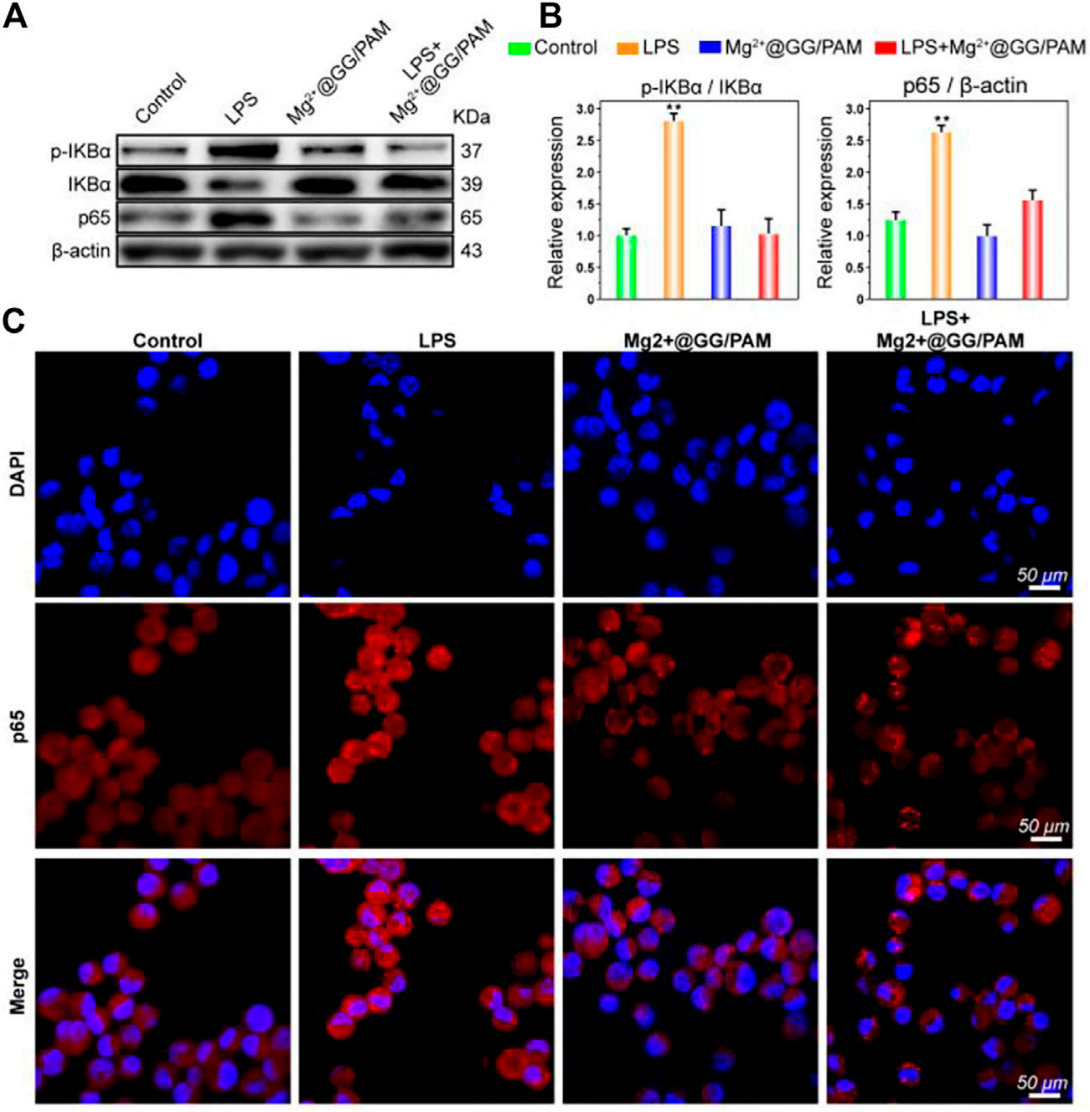
The protein expression of NF-κB p65 was detected by Western blotting after treatment with Mg^2+^@GG/PAM hydrogel and LPS **(A)**. The statistical analysis of *p*-IKBα, IKBα, and NF-κB p65 expression (means ± SD, *n* = 4). **(B)** RAW264.7 cells were stained with anti-p65 antibody and DAPI after culturing onto control, GG, GG/PAM, and Mg^2+^@GG/PAM hydrogel for 72 h and visualized by fluorescence microscopy. **(C)** *p < 0.05, **p < 0.01 vs control.

An inflammatory response is critical for skin homeostasis reconstruction after burn injury, and this directly affects fibroblast proliferation and wound healing efficiency. In fact, Mg^2+^ ions serving as a protective agent have been applied in neuroprotection, preeclampsia, and preterm labor fields in clinics (Duley et al., 2010; Crowther et al., 2017). There is abundant molecular-level evidence demonstrating that Mg^2+^ ions could restrain macrophages generating pro-inflammatory cytokines TNF-α and IL-6 (Lin et al., 2010). Son et al. further report that the increased Mg^2+^ ions could suppress the production of reactive oxygen species (ROS) and NO in immune cells (Son et al., 2007). The results suggest that Mg^2+^ ions serving as a novel anti-inflammatory agent play a role in eliminating excessive inflammation for immune protection. Similarly, Mg-related biomaterials also have the potential to perform anti-inflammatory functions. To verify the anti-inflammatory action of Mg^2+^@GG/PAM hydrogel, we investigated the effect on the traditional NF-κB pathway, which is a transcription factor typically associated with inflammation and infection. To verify the anti-inflammation function, the macrophages were cultured onto the hydrogel surface with LPS added. In Figures 5A,B, the LPS stirs up the expression of phosphorylation-IKBα and p65. Nevertheless, when RAW264.7 cells were cultured onto the Mg^2+^@GG/PAM hydrogel surface, the phosphorylation-IKBα and p65 protein levels were suppressed, indicating that the Mg^2+^@GG/PAM hydrogel might inhibit inflammation via the inhibition of NF-κB p65 protein expression. This result is in accordance with the research of Christineet et al. confirming that MgSO_4_ served as an anti-inflammatory agent that inhibited endothelial cell activation via the NF-κB pathway during preterm labor (Rochelson et al., 2007). To future validate the NF-κB pathway inhibition effect of Mg^2+^ ions in a visual way, the immunofluorescence of p65 protein in RAW264.7 cells is shown in Figure 5C; LPS could increase the aggregation of p65 protein in the nucleus although, when the RAW264.7 cells are cultured on the Mg^2+^@GG/PAM hydrogel surface, the amount of p65 was decreased, indicating that Mg^2+^ ions could inhibit the NF-κB pathway brought by LPS. These results were favorable for Mg^2+^@GG/PAM hydrogel to reduce inflammation in the burn healing period.

### *In Vivo* Healing-Impaired Burn Wound Model in Rats

**FIGURE 6:**
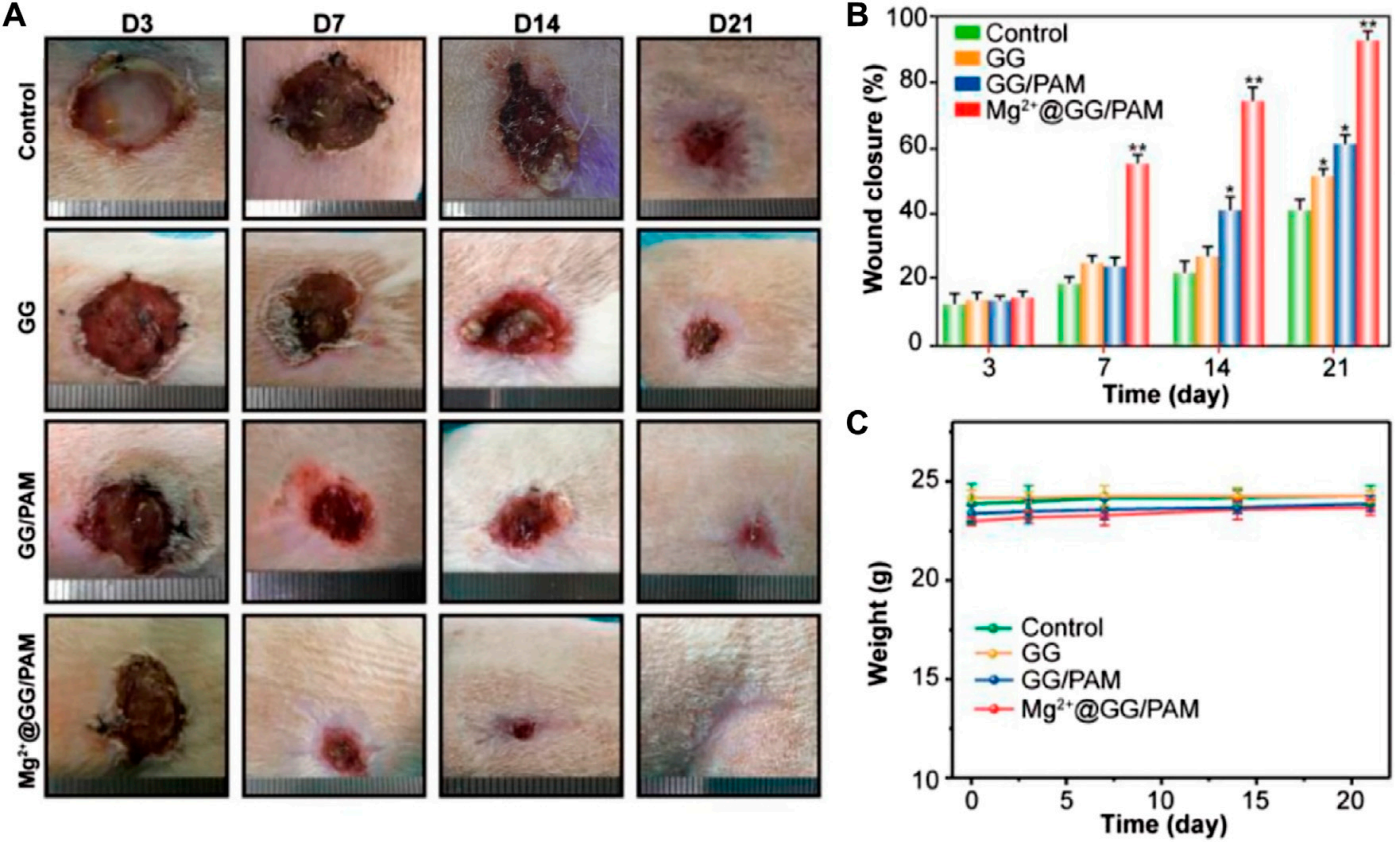
Macroscopic observation **(A)**, statistical analysis **(B)**, and weight changes **(C)** of wound healing process at 3, 7, 14, and 21 days, after treatment with PBS (control), GG, GG/PAM, and Mg^2+^@GG/PAM. The values are represented as mean ± SD (*n* = 6). *p < 0.05, **p < 0.01 vs control.

The burn wound healing efficiency of the composite hydrogels was evaluated in a rat burn wound model. The rats’ burn wounds were treated with PBS, GG, GG/PAM, and Mg^2+^@GG/PAM hydrogel, respectively. The wounds were created for nummular shaping at about 18 mm in diameter. At regular intervals, the wound healing status is shown in Figure 6A. The change of diameter of the wounds (Figure 6B) and body weight (Figure 6C) were recorded at regular times. For the Mg^2+^@GG/PAM hydrogel group, the burn wound size decreased obviously and achieved complete reconstruction at the 21st day with no scar and fully covered with hair. The Mg^2+^@GG/PAM hydrogel group has the fastest wound closure speed compared with other groups. For the PBS group, despite saline slightly accelerating the healing process for the contraction function, the full-thickness burn wound closure rate is only about 45% at the 21st day (Svensjö et al., 2000). For the GG and GG/PAM hydrogel groups, the wound healing speed was faster than the PBS group. This may be because the GG hydrogel matrix could maintain the wound’s moisture, absorb excess exudate, cover the sensitive underlying tissue, and thus provide a similar extracellular matrix environment for cell growth and proliferation (Lee et al., 2003). Foremost, Mg^2+^@GG/PAM hydrogel not only provided a good 3-dimensional environment for cell proliferation, it also served as an Mg^2+^ ion delivery system to the wound area. The continuously released Mg^2+^ ions play a crucial role in burn wound repair. Thus, Mg^2+^@GG/PAM hydrogel achieved the fastest burn healing rate in a bioactive way.

### Histological Observations on Repair of Burn Wound Model

**FIGURE 7:**
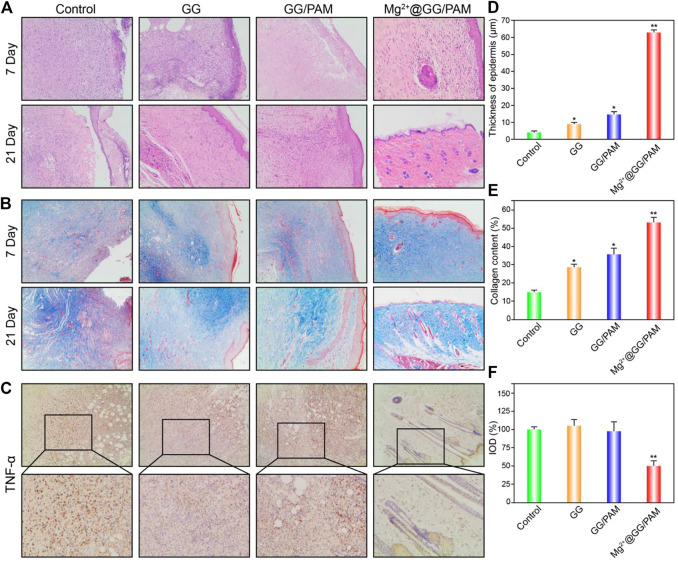
Histological evaluation of the wound at 7 and 21 days using hematoxylin and eosin (HE) staining **(A)**, Masson’s trichrome staining **(B)**, and TNF-α immunohistochemical staining **(C)**, thickness of epidermis statistics **(D)** and collagen content statistics via ImageJ software **(E)**. Integrated optical density of TNF-α was quantified on immunohistochemistry slices **(F)**. The values are represented as mean ± SD (*n* = 6). *p < 0.05, **p < 0.01 vs control.

To better observe the burn wound healing effect of Mg^2+^@GG/PAM hydrogel, hematoxylin and eosin (HE), Masson’s trichrome, and TNF-α immunohistochemical staining were carried out. For the HE staining (Figure 7A), on the 7th day, wounds in the control group (no dressing) and pure GG dressing group did not form the cuticle structure, whereas the GG/PAM and Mg^2+^@GG/PAM groups formed thin cuticles. On the 14th day, skin appendages could be clearly observed in the Mg^2+^@GG/PAM group, which possesses a significantly higher number of dermal appendages, such as hair follicles, than the other three groups, indicating that the released Mg^2+^ ions prominently facilitated the burn wound healing effect. For the Masson staining (Figure 7B), on the 7th day, collagen distribution at the wound area was disorganized in all groups except for the Mg^2+^@GG/PAM group. Besides this, the few hair follicles were regenerated in the Mg^2+^@GG/PAM group. On the 21st day, the blue color was deeper for each group. Nevertheless, the collagen fibers in the control group and pure GG group are still scattered, but the GG/PAM and Mg^2+^@GG/PAM group collagen distribution was more uniform. Besides this, to assess the inflammation level in the wound, TNF-α was selected as an indicator of the inflammatory level. TNF-α is mainly secreted by mononuclear macrophages, which is a downstream protein of the NF-κB pathway and could inhibit the wound healing effect by provoking the immune cells to release somatostatin. It can be seen from Figure 7C that the Mg^2+^@GG/PAM group represents the lowest TNF-α expression on the 21st day, suggesting that the released Mg^2+^ ions could reduce the inflammation and further accelerate burn wound healing. This result was consistent with the *in vitro* result that Mg^2+^@GG/PAM hydrogel can inhibit the NF-κB pathway in RAW264.7 cells. In addition, the epidermal thickness, collagen content, and TNF-α expression were quantificational. It can be seen from Figure 7D that the epidermal thickness was ∼61 μm in the Mg^2+^@GG/PAM hydrogel group, which was significantly thicker than the other groups. The collagen content is also highest in the Mg^2+^@GG/PAM hydrogel group, indicating better healing quality in the case of the Mg^2+^ ions (Figure 7E). As shown in Figure 7F, the Mg^2+^@GG/PAM group revealed the lowest expression quantity of TNF-α on the 21st day compared with the other groups. These results indicate that Mg^2+^@GG/PAM hydrogel promotes burn wound healing by inducing angiogenesis inflammation inhibition. All these results indicate that Mg^2+^@GG/PAM hydrogel is an effective dressing for burn wound healing, exhibiting collagen maturation and fewer inflammation properties.

## Conclusion

In summary, we prepared hydrogels consisting of an ionically crosslinked GG biopolymer and a covalently crosslinked synthetic polymer, poly (acrylamide). These gels displayed double network behavior for improved mechanical properties compared with their respective single network hydrogels. The INP hydrogels show that the tension strength of Mg^2+^@PAM/GG hydrogel is obviously increased from 86 to 392 MPa, the elongation at break increased from 84 to 231%, and crosslinking density N increased from 4.3 to 7.2 mol/m^3^. We suggest that this favorable mechanical behavior can be attributed to the reversible characteristics of the ionically crosslinked biopolymer network in the INP hydrogel. The *in vitro* experiments suggest that Mg^2+^@PAM/GG hydrogel possesses good biocompatibility and proliferation for fibroblast cells and NF-κB pathway inhibition ability for RAW264.7 cells. In an *in vivo* full-thickness burn wound rat model, Mg^2+^@PAM/GG hydrogel accelerated the healing rate of burn wounds at 21 days compared with the other groups. These results suggest that Mg^2+^@PAM/GG hydrogel is a promising wound dressing for healing full-thickness burn wounds.

## Data Availability

The original contributions presented in the study are included in the article/Supplementary Material, further inquiries can be directed to the corresponding authors.
