# Corrigendum: The fabrication of a gellan gum-based hydrogel loaded with magnesium ions for the synergistic promotion of skin wound healing

**DOI:** 10.3389/fbioe.2023.1335918

**Published:** 2023-12-14

**Authors:** Wenqiang Li, Xingling Jian, Yanfen Zou, Lin Wu, Haiyan Huang, Hui Li, Dandan Hu, Bo Yu

**Affiliations:** ^1^ Department of Dermatology, Skin Research Institute of Peking University Shenzhen Hospital, Peking University Shenzhen Hospital, Shenzhen, China; ^2^ Guangdong Provincial Engineering Technology Research Center for Sports Assistive Devices, Guangzhou Sport University, Guangzhou, China; ^3^ Child Healthcare Department, Guangzhou Women’s and Children’s Medical Center, Guangzhou Medical University, Guangzhou, China

**Keywords:** gellan gum, magnesium ion, polyacrylamide, skin wounds, hydrogel

## Abstract

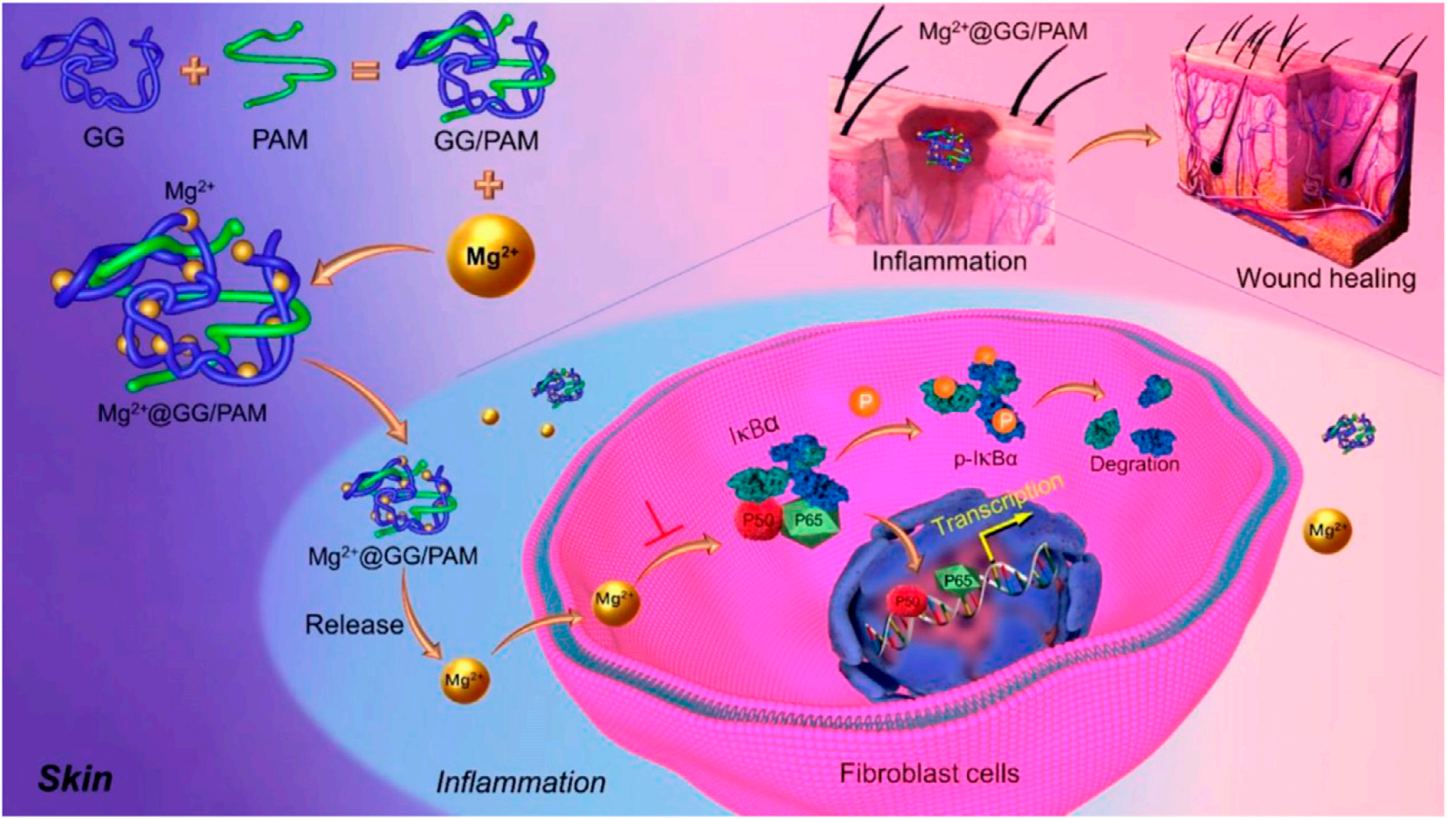

In the published article, there was an error in **Affiliation** 2. Instead of “Gungdong provincial engineering technology research center for sports assistive devices, Guangzhou Sport University, Guangzhou, China.”, it should be “Guangdong Provincial Engineering Technology Research Center for Sports Assistive Devices, Guangzhou Sport University, Guangzhou, China.”

In the published article, there was an error in the order of “**Graphical Abstract**, Scheme 1, and Figure 1”, and in the legend for “Scheme 1, Figure 1” as published. The corrected order and legend appears below.

**SCHEME 1:**
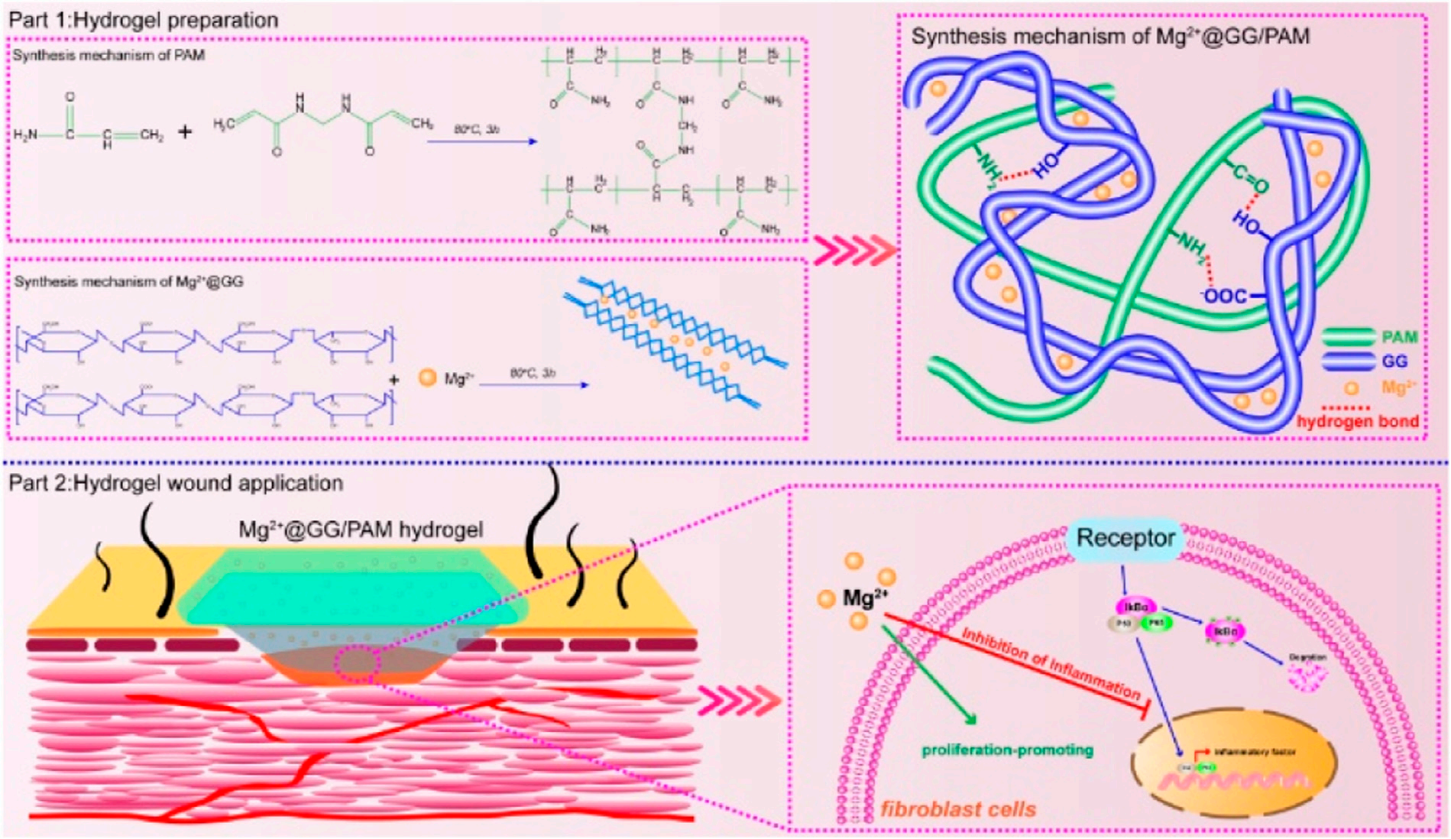
Schematic illustration of synthesis procedure for Mg^2+^@GG/PAM hydrogel and the repair mechanism of Mg^2+^ ions from Mg^2+^@GG/PAM hydrogel in the burn wound.

**FIGURE 1:**
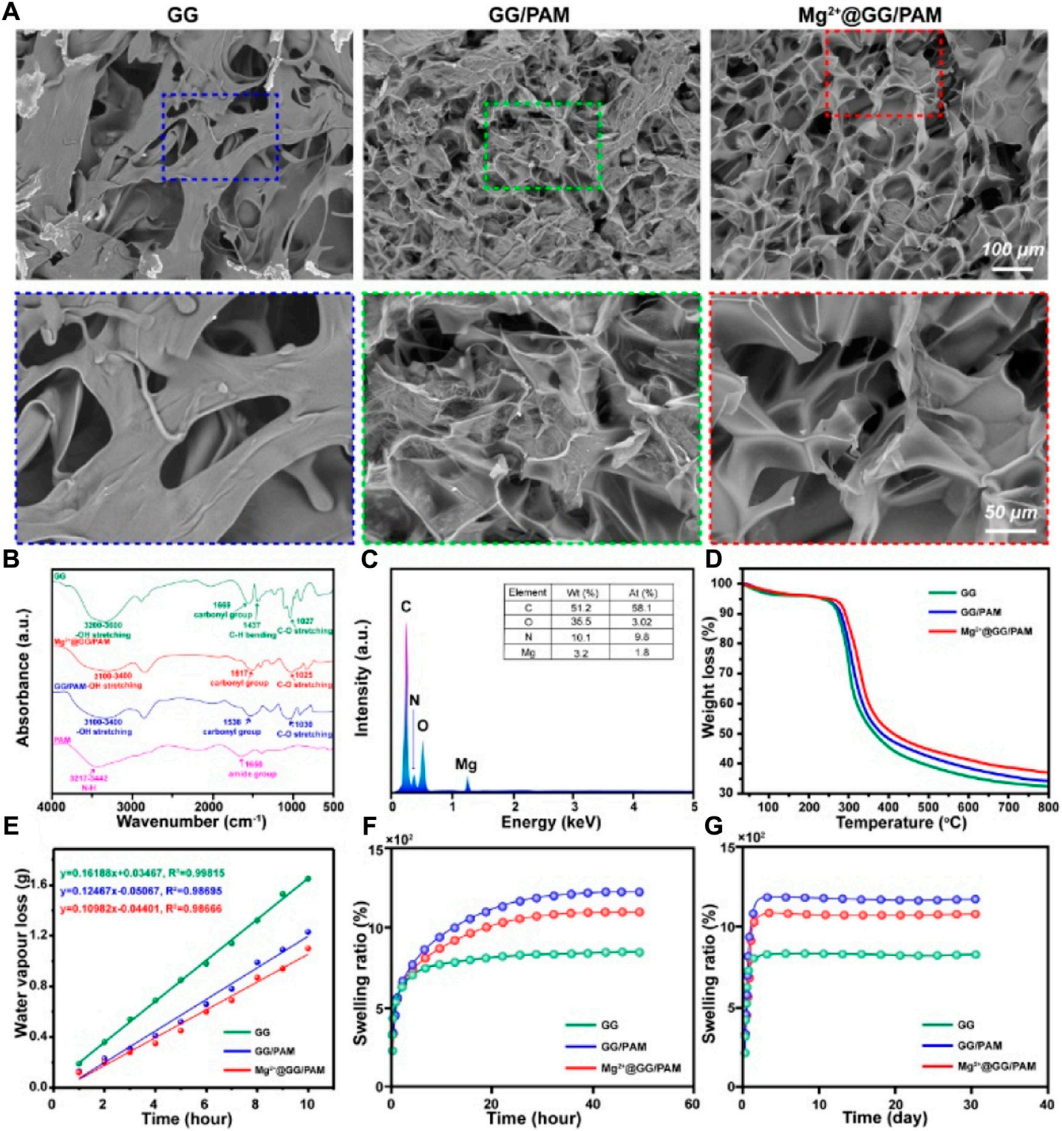
Characterization of Mg^2+^@GG/PAM hydrogel **(A)** SEM **(B)** FT-IR **(C)** EDS analysis **(D)** TGA **(E)** Water vapor transmission rate **(F–G)** Swelling ratio.

In the published article, there was an error in Figure 6 as published. The corrected Figure 6 and its caption appear below.

**FIGURE 6:**
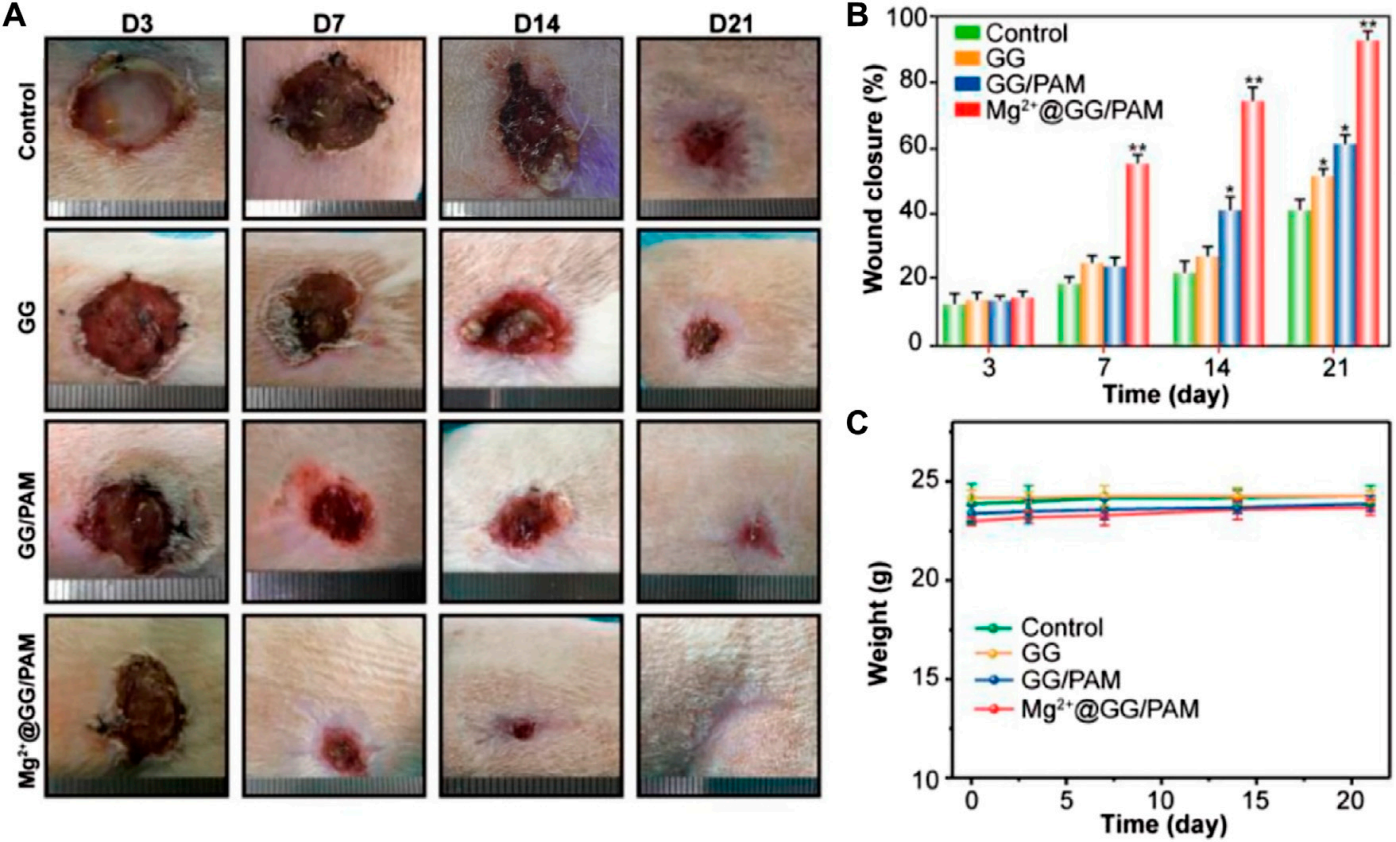
Macroscopic observation **(A)**, statistical analysis **(B)**, and weight changes **(C)** of wound healing process at 3, 7, 14, and 21 days, after treatment with PBS (control), GG, GG/PAM, and Mg2^+^@GG/PAM. The values are represented as mean ± SD (*n* = 6). *p < 0.05, **p < 0.01 vs control.

The authors apologize for these error and state that this does not change the scientific conclusions of the article in any way. The original article has been updated.

